# Breaking Down Complications With Locoregional Anesthesia: A Game-Changer for Pain Management in Pediatric Emergencies

**DOI:** 10.7759/cureus.75299

**Published:** 2024-12-07

**Authors:** Luca Gentili, Paolo Scimia, Massimiliano Luca D'Agostino, Antonio De Cato, Alberto Pasqualucci, Giustino Varrassi, Chiara Angeletti

**Affiliations:** 1 Anesthesia and Intensive Care Unit, Santa Maria Goretti Hospital, Latina, ITA; 2 Anesthesia and Intensive Care Unit, Giuseppe Mazzini Hospital, Teramo, ITA; 3 Clinical Medicine, Public Health and Life Science (MESVA), University of L'Aquila, L'Aquila, ITA; 4 Anesthesia and Critical Care, University of Perugia, Perugia, ITA; 5 Pain Medicine, Fondazione Paolo Procacci, Rome, ITA

**Keywords:** emergency surgery, fascial plane blocks, locoregional anesthesia, non-pediatric settings, pediatric anesthesia, postoperative pain management

## Abstract

The management of postoperative pain in pediatric patients undergoing emergency surgical procedures, particularly in non-pediatric hospitals, presents significant challenges due to the unique physiological requirements of children. The utilization of opioid analgesia may result in severe complications, necessitating a transition toward multimodal analgesia, which integrates various pain management strategies to enhance effectiveness while mitigating adverse effects. Locoregional anesthesia techniques, such as fascial plane blocks, provide targeted pain alleviation, reducing dependence on opioids. Recent advancements in ultrasound-guided methodologies have markedly improved safety and precision in this context. This report presents two cases involving pediatric patients aged eight and 12 years who underwent urgent posttraumatic open splenectomy. Both patients exhibited stable hemodynamic parameters and had no significant prior medical history. Following surgery, they received an ultrasound-guided rectus sheath block (RSB) and dynamic transversus abdominis plane blocks (TAPBs) utilizing a mixture of ropivacaine, dexamethasone, and clonidine. Fentanyl was administered before and during the surgical procedures, which lasted approximately 75 minutes. Upon regaining consciousness, both patients indicated a visual analog scale (VAS) pain score of 0. They required only a single dose of intravenous acetaminophen for pain relief, demonstrating effective opioid-free pain management and achieving a high level of parental satisfaction. Combined RSB and TAPB provide adequate and safe postoperative pain management for pediatric patients undergoing emergency splenectomy in a non-pediatric hospital setting. This approach can reduce opioid dependence and improve patient outcomes. Further research is warranted to explore the broader application of locoregional anesthesia techniques for pediatric emergency surgery in non-pediatric settings.

## Introduction

The management of postoperative pain in pediatric patients undergoing emergency surgery is a peculiar challenge that necessitates early intervention. In contrast to adults, children possess unique physiological and anatomical traits that require a customized method for managing pain. Opioid-based analgesia, while effective, carries the risk of complications such as respiratory depression, nausea, vomiting, and opioid-induced constipation (OIC) [1]. Multimodal analgesia is a fundamental principle of pain management, also in children, according to which drugs and techniques targeted at different mechanisms of providing analgesia, transduction, and perception of nociceptive stimuli should be combined to achieve desirable results. However, using systemic drugs has potential complications and side effects. In multimodal analgesia, the combined impact of various analgesics, potentially with a synergistic effect, helps achieve sufficient pain relief at a lower dosage of each drug, with reduced side effects. Integrating analgesics with regional or locoregional techniques can enhance this effect [2].

Anesthesiologists operating within non-pediatric hospitals may have limited experience in the management of postoperative pain among pediatric patients. This can lead to a reluctance to administer opioids due to concerns about potential complications, such as opioid-induced respiratory depression and/or OIC, which can be particularly serious in children [3]. Opioid underdose to prevent these complications can result in inadequate pain control, hindering patient recovery and exposing them to the risk of developing chronic pain. Recent studies indicate that children may experience prolonged pain following major surgery, like adults, which can seriously impact their health outcomes [4]. Locoregional anesthesia offers a compelling solution to this dilemma. By providing targeted pain relief specific to the surgical site, it minimizes the need for systemic analgesics and their associated side effects.

Regional anesthesia (RA) techniques include neuraxial blocks (e.g., caudal blocks (CBs)), peripheral nerve blocks (PNBs), and fascial plane blocks (FBPs). The anatomy of injection sites and doses varies significantly for pediatric patients, from neonates to adolescents. Ultrasound support may improve the quality, effectiveness, and safety of this analgesia. Pediatric RA has significantly expanded over the last decade, becoming integral to medical practice. More enhanced recovery after surgery (ERAS) protocols are keys to improving postoperative outcomes in children and reducing hospital stays. However, RA may still pose risks such as local anesthetic systemic toxicity (LAST), nerve injury, bleeding, and infection.

In pediatric surgeries, few FBPs used in adults are relevant. Common pediatric FPBs include rectus sheath block (RSB), transversus abdominis plane (TAP) block (TAPB), quadratus lumborum (QL) block (QLB), transmuscular QLB (TQLB), and erector spinae plane block (ESPB). Literature indicates that central and PNBs offer superior, longer-lasting, and safer postoperative pain control than systemic analgesia, serving as a valid alternative for minor to moderate surgery [5]. Fascial blocks are infrequently used for postoperative pain management during emergency surgery in pediatric patients at non-pediatric hospitals. However, the expertise accumulated in administering RA in adult populations [6] and the requisite technical skills suggest that these methodologies may also be suitable for pediatric surgical cases in non-specialized environments.

We present our experience with two pediatric patients necessitating emergency posttraumatic splenectomy at a non-pediatric facility. Postoperative pain management was obtained with the RSB alongside the dynamic TAPB. This report, to our knowledge, is unique in combining these two fascial blocks for postoperative pain management in a non-pediatric context.

## Case presentation

We present two cases of pediatric patients (an eight-year-old boy, 27 kg, 122 cm, and a 12-year-old boy, 40 kg, 145 cm) who underwent urgent posttraumatic open splenectomy. Both patients had no prior medical history of pathologies or surgeries. No pre-emptive analgesia was administered. Before and during surgery, hemodynamic parameters remained stable. Following surgery, a bilateral ultrasound-guided RSB and dynamic TAPB were performed following the technique described by Scimia et al. [6], reaching the lateral surface of the QL muscle with the tip of the needle. For both fascial blocks, an anesthetic solution composed of ropivacaine 0.125% with dexamethasone 1 mg and clonidine 1 mcg/kg for each puncture (total dexamethasone: 4 mg; total clonidine: 27 mcg and 40 mcg, respectively) was administered. The volume used was 15 mL (18.75 mg of local anesthetic) per side for the RSB and 20 mL (25 mg of local anesthetic) per side for the TAPB. Before the induction of general anesthesia (GA), fentanyl 2 mcg/kg was administered, followed by a single bolus of 3 mcg/kg during surgery. The duration of surgery was approximately 75 minutes for both cases. Upon awakening, the visual analog scale (VAS) pain score was 0 for both patients. Throughout the entire follow-up period (six, 12, 24, 36, and 72 hours), each patient only required a single dose of paracetamol 15 mg/kg intravenously. In these cases, RA for postoperative pain management effectively avoided the use of opioids and their associated side effects. Recovery was free of complications, and parents reported high satisfaction with the anesthetic strategy, due to their children experiencing minimal pain after the accident and surgery. Informed consent was obtained from relatives.

## Discussion

RA has become a cornerstone of perioperative pain management in pediatric surgery, offering numerous benefits over traditional opioid-based approaches. FPBs have emerged as particularly effective techniques for providing both intraoperative and postoperative analgesia, reducing opioid requirements, and improving patient outcomes [7]. While opioids are analgesics commonly used in children, mainly for relieving moderate to severe pain, an increasing number of reports point to adverse aspects of opioid effects: immunosuppressive effects, possible opioid-induced hyperalgesia, or increased tolerance to opioids [2].

A review of orthopedic oncologic surgeries highlighted the efficacy of RA in mitigating postoperative pain. However, the authors emphasized the importance of a multimodal approach, combining RA with other pain management strategies [8]. RA techniques within multimodal plans have consistently demonstrated their ability to reduce severe immediate postoperative pain, a significant risk factor for chronic postsurgical pain (CPSP) [9].

There is growing evidence suggesting that optimized analgesia in the acute postoperative period can help prevent the development of chronic pain [10]. For patients at high risk of chronic pain (e.g., laparoscopic abdominal surgery, breast cancer surgery, and thoracotomies), early initiation of RA before incision, followed by continuous administration for 24 to 72 hours, is recommended as a preventative strategy [11].

Ultrasound technology has granted a significant evolution in RA, enabling the accurate and precise performance of various FPBs. Ultrasound-guided FPBs have significantly improved pain management for chest and abdominal wall surgeries, expanding the scope of pediatric anesthesia [12]. Ultrasound has increased the success rate of these blocks, reduced the required dosage of local anesthetics, and allowed visualization of the spread of the anesthetic along the desired fascial planes [13].

Although many FPBs have been outlined for adults, only a limited number are typically utilized in pediatric surgery because of anatomical variations [14]. The most commonly employed FPBs are the RSB, TAPB, and QLB. The RSB is frequently employed to provide postoperative pain relief for surgeries involving midline incisions, such as umbilical hernia repair and single-incision laparoscopic surgery. Studies have demonstrated the effectiveness of this block in reducing perioperative pain in pediatric patients undergoing umbilical hernia repair [15]. However, the depth of the posterior rectus sheath can vary, making ultrasound guidance essential for successful block placement.

TAPBs are used for analgesia in surgeries of the anterolateral abdominal wall. While they provide somatic analgesia, they do not address visceral pain. In a study of children undergoing open appendectomy, unilateral TAPBs significantly reduced morphine consumption in the first 48 hours postoperatively [16]. Visceral postoperative pain may also be treated with the use of intraperitoneal local anesthetics [17].

In pediatric anesthesia, CB has long been a pain management practice. A recent meta-analysis revealed that TAPBs and CBs provide similar early analgesia and safety profiles in children undergoing abdominal surgeries [18]. Additionally, no differences were observed in adverse effects between them.

The QLB is a relatively novel, deep FPB that has recently gained popularity. The QL muscle lies in the posterior abdominal wall, and the thoracolumbar fascia (TLF) covers it. The TLF is crucial in spreading local anesthetic into the thoracic paravertebral space and contains a high density of sympathetic fibers and mechanoreceptors. One advantage of the QLB is its ability to provide both somatic and visceral analgesia, making it an effective analgesic tool for abdominal surgery [19-21].

In previous meta-analyses, the QLB has demonstrated effective pain relief in adults [22]. However, children typically experience more robust pain responses than adults, and there are limited options for managing pediatric pain. Zhao et al. [22] recommended the use of QLB for pediatric patients undergoing lower abdominal surgery due to its effectiveness as a postoperative analgesic technique despite limited research evidence. This is confirmed by a recent network meta-analysis by Hung et al. [23]. The QL and TAP blocks provided the longest time to first rescue analgesia and reduced analgesic needs in pediatric inguinal surgeries. The QLB mainly had the best duration for hernia repairs, while the CB uniquely extended the time to first rescue analgesia during orchidopexy.

In a recent randomized clinical trial, Zhang et al. [24] found that QLB provided more effective postoperative pain relief for children undergoing laparoscopic abdominal surgery compared to TAPB and CB. A growing trend in locoregional anesthesia is the combination of two or more anesthetic blocks to achieve a synergistic analgesic effect and increase dermatomal coverage. The association of ilioinguinal-iliohypogastric (II-IH) and TAPB can represent a scarcely invasive and reasonable alternative to caudal analgesia in pediatric genitourinary surgery [5]. As demonstrated by Priyadarshini et al. [25], QLB provides a prolonged period of analgesia and leads to decreased opioid consumption compared with TAP blocks and II-IH nerve blocks in children undergoing open inguinal hernia repair.

In our clinical practice, this combined approach has facilitated opioid-free postoperative pain management in a non-pediatric hospital setting. Several case reports have documented the successful application of combined fascial blocks, demonstrating their synergistic action in covering the entire surgical area [6,26,27].

A multimodal approach, incorporating regional analgesia techniques like caudal epidural blockade alongside non-pharmacological strategies (physical therapy, heat therapy, and psychological support), has been recognized for improving pain management and reducing side effects in light of recent ERAS protocols [28]. The implementation of ERAS protocols has progressively reduced opioid usage, leading to better pain control, earlier ambulation, and shorter hospital stays, ultimately improving patient outcomes.

RA should be strongly considered for improved patient outcomes, particularly in pediatric emergencies. Pain evaluation in children can be challenging, and their narrow and variable drug therapeutic index necessitates a cautious approach to medication use [29]. As highlighted by Gill et al. [3], opioid-induced respiratory depression, while infrequent, can have severe consequences in pediatric patients. A recent meta-analysis comparing apnea episodes between GA and regional plus GA showed a higher incidence of apnea in the GA group [30]. Safe opioid use in this population requires careful consideration of the dosage, route, and administration method, as well as attention to predisposing factors like reduced respiratory reserve and underlying medical conditions [3,31].

Therefore, it is essential to standardize training and ensure that crucial skills are developed for safe practice, improving patient care and outcomes, even in non-pediatric settings. RA experts recently produced a core curriculum to standardize training and ensure essential skills for safe practice, thus improving patient care and outcomes [32]. As demonstrated in this experience, knowledge of locoregional techniques has also enabled optimal management of emergency, open pediatric surgery, and postoperative pain in a non-pediatric hospital.

## Conclusions

In conclusion, these case reports underscore the efficacy and safety of locoregional anesthesia as a valuable tool for managing postoperative pain in pediatric emergency surgery within non-pediatric hospitals. The combined RSB and bilateral dynamic TAPB effectively reduced opioid use and its associated side effects in our patients. This approach improved patient outcomes and facilitated a smoother recovery process. The broader implication lies in the potential of locoregional anesthesia to bridge the gap in pain management for pediatric emergencies within non-pediatric settings. By offering targeted pain relief and minimizing the risk of opioid-related complications, locoregional anesthesia can enhance patient care, potentially streamline resource allocation, and ensure adequate pain control for these vulnerable patients. Further research is warranted to explore the broader application of locoregional anesthesia techniques for pediatric emergency surgery in non-pediatric settings.
